# ‘Playlist for Life’ at the end of life: a mixed-methods feasibility study of a personalised music listening intervention in the hospice setting

**DOI:** 10.1186/s40814-022-00983-8

**Published:** 2022-02-07

**Authors:** Bridget Johnston, Fiona Bowman, Emma Carduff, Fulya Donmez, Andy Lowndes, Alistair McKeown

**Affiliations:** 1 School of Medicine, Dentistry and Nursing, University of Glasgow, Glasgow, Scotland, UK; 2 NHS Greater Glasgow and Clyde, Glasgow, Scotland, UK; 3 Marie Curie Hospice, Glasgow, Scotland, UK; 4 Playlist for Life Registered Charity, SC044072 Glasgow, Scotland, UK; 5 The Prince and Princess of Wales Hospice, Glasgow, Scotland, UK

**Keywords:** Feasibility study, End-of-life care, Music, Palliative care, Hospice

## Abstract

**Background:**

Playlist for Life is a brief, inexpensive music listening intervention which originated in dementia care, but is increasingly being used for people at the end of life. However, there is a lack of robust empirical research on its application in the hospice setting. Our patient and public involvement group originated the idea for this study. The aim of this feasibility study was to inform the design of a larger effectiveness study on the use of Playlist for Life in the hospice setting.

**Method:**

This study was a mixed-methods feasibility study involving adults at the end of life, family members and hospice staff from one in-patient hospice in Scotland. Eligible patient/family member dyads were approached by hospice staff and if interested, recruited by the researcher. All included participants received the intervention, which involved the provision of an MP3 player and assistance to set up a playlist. Participants were asked to listen to the playlist daily during the intervention period (7 days). Data were collected through patient reported outcome measures and on days 1, 3 and 7 of the intervention period and through participant observation session. Patient/family member dyads and hospice staff also took part in qualitative interviews (Appendix 1) post-intervention, which were audio-recorded, transcribed and analysed thematically. Semi-structured interviews at the end of the intervention period were used to evaluate feasibility and acceptability. An advisory group including patients, family members and staff gave helpful feedback on the qualitative interview questions. Interview questions were the same for all participants and all the questions were asked to all participants.

**Results:**

*N* = 15 participants were recruited (*n* = 5 patients, *n* = 5 family, *n* = 5 staff. The intervention was appraised positively, particularly regarding its beneficial effect on patient/family relationships. The study design was deemed feasible and acceptable.

**Conclusion:**

The findings of this study will inform the development of a future randomised cluster trial designed to assess the usability and effectiveness of the Playlist for Life personalised music intervention.

****Trial registration**:**

This study was not registered as this was a small feasibility study, conducted prior to a pilot study not testing for effectiveness. In addition, the study was non-randomised. The study is registered with NHS ethics and the hospice research and governance team

## Background

In Scotland, around 54,000 people die every year and over 200,000 people are particularly affected by the death of significant individuals. In general, the deaths occur in older age, sometimes accompanied by frailty, dementia and multiple other conditions. It is estimated that by 2037 the number of people dying each year will have risen by 12% to 61,600. It is estimated that up to 8 out of 10 individuals who die have needs that would benefit from the provision of palliative care [1]. A key aim of palliative and end of life care is to ensure holistic care and support is aligned to the needs and preferences of the dying person, their families and carers [2].

This study originated from discussion with our public involvement group and clinical partners. The group has been strongly supportive of the Playlist for Life intervention which is based on individuals’ personal history and music preferences, and the wider need to build a rigorous evidence base for interventions employed in end of life care [3–9]. Playlist for Life is a brief and inexpensive music-based intervention that can promote shared connections, personhood and legacy. It is an intervention founded by the writer and TV presenter Sally Magnusson in memory of her mother, Mamie. When Mamie lost her memory, Sally found that personally meaningful music helped improve Mamie’s quality of life more than anything else. Playlist for Life is based on the idea that sharing personalised musical experiences can promote communication and enrich the bond between the person receiving care and their loved ones [10, 11]. Although the origins are in dementia care, Playlist for Life has been introduced within inpatient hospice and hospital settings to support people at the end of life and to support patients and their families at the end of life, as well as help relieve anxiety, distress, pain and other physical symptoms [2, 12, 13].

Everyone has a soundtrack of their life: special songs and music that bring back memories. Listening to these can help to coordinate neural activity [14]. The synthesis of music, emotion and autobiographical memories within the pre-frontal cortex appears to improve mood, promote awareness and support memory retrieval and personhood [2, 13]. Indeed it is suggested, in dementia, the brain continues to interpret and understand musical input when other areas of functional ability have deteriorated [15].

Music therapy and listening to music are two techniques which come under the broad theme of music interventions. Music therapy requires specific training for music therapists and theoretical–methodological support and focuses on the role of music elements in the relationship between the patient and the therapist. However, listening to music is not based on a relationship between patient and music therapist [16]. While both interventions can be person centred, music therapy requires an accredited and qualified professional to undertake music intervention sessions which can be live and improvised involving a wide range of music styles [16]. Conversely, listening to music involves sessions using purposefully chosen pre-recorded songs or tracks [17]. Additionally, listening to music, as with the Playlist for Life intervention, can be repeated and the song list kept and reused as desired. Playlist for Life can be described as a more relaxing method of music intervention as research has found music listening, compared to music therapy, offers the comfort of hearing familiar songs and the security of listening through headphones enabling the person to solely concentrate on the audio [18]. Moreover, a crossover study on the effect of music interventions (listening to music and music therapy) on behavioural and psychological symptoms of dementia has reported that comparing listening to music and music therapy showed no significant statistical differences [16].

Our focus was on the personalised nature of the intervention (i.e. music of the person’s own choosing that connects with, or speaks to, memorable moments in their lives or aligns with their musical preferences) and the quality and impact of the shared interaction that occurs between the person and their family member when using Playlist for Life together. Underpinning this, and in contrast to much existing research, was a unique focus on the role of Playlist for Life, where we were interested in enhancing personhood and legacy for people at the end of life.

Overall, research into personalised music intervention is in its infancy but seminal work within dementia care points to its value in enhancing life quality and stimulating autobiographical memory [19–25]. Listening to music evokes vivid and emotional memories of events from across the lifespan by activating the limbic system. More recent evidence demonstrated that songs are strongly linked to memories of a person, period, or place [21–25]. Previous literature highlighted cited benefits were enhanced mood, increased awareness of other people and surroundings, improved cognitive capacity and a greater sense of identity and independence [26–28]. Moreover, another study for older adults with dementia and symptoms of agitation based on the individualised music protocol of Gerdner and colleagues’ highlighted that agitation was decreased while participants were listening to music compared to times when they were not listening to music [29, 30]. Other advantages are that listening to music is low cost, is relatively straightforward to deliver, and is sustainable over the longer term, indicators that translate well for wider use [31]. Clinical studies have demonstrated the effectiveness of music interventions in decreasing symptoms of depression and anxiety for people who are approaching end of life [32–39]. Furthermore, a systematic review and meta-analysis results showed that music therapy had an overall medium-to-large effect on stress-related outcomes [40]. Another systematic review on music intervention in palliative and end-of-life care emphasised that music intervention has indicated significant benefit in respect to psychological, physiological and social responses [41–43]. In addition, a recent Cochrane review examining music interventions for improving psychological and physical outcomes in patients with cancer identified that listening to music alleviated pain, and music interventions had beneficial effects on a range of symptoms and physiological markers such as depression and anxiety [17]. Other studies suggested some evidence for music in pain relief [36]. Current evidence indicates, however, that there is a lack of robust evaluation of evidence-based music interventions using specific measures designed for the terminally ill and which evaluate specific music modalities in particular settings across multiple sessions [7, 44, 45].

To date, there has been no empirical research demonstrating whether, and in what ways, patient and carer participation in Playlist for Life has an impact on patients with palliative care needs at the end of life [46, 47]. Our principal objective was to undertake feasibility work to determine the acceptability of Playlist for Life implementation in the hospice setting. The findings of this proposed study can establish whether Playlist for Life is a feasible and acceptable intervention for people at the end of their life and their families and can help to inform the design and evaluation of Playlist for Life in a future definitive randomised controlled trial.

## Methods

### Study aims

The primary aim of this study was to evaluate the feasibility and acceptability of Playlist for Life for adults at the end of life, family members and hospice staff in the hospice setting in Scotland.

Objectives were to (i) determine the most acceptable and appropriate format for patient/carer delivery of the Playlist for Life intervention within the context of palliative and end of life hospice-based care, (ii) assess whether our research questions for the qualitative component provide rich, descriptive data on participant experience, and (iii) identify whether this study is acceptable to develop a standardised intervention package that is suitable for implementation in a future randomised controlled trial.

### Study design and procedure

This study was a single-arm, non-controlled, mixed-methods feasibility study and an embedded qualitative study underpinned by the pragmatic paradigm [48]. We followed the Consort reporting guideline [49] and MORE CARE statement [50] for developing research interventions in end of life care. Feasibility studies provide a valuable contribution to research, the results can prove useful benchmarks in the subject area regardless of meeting the original objectives [51]. Given that our aim was to evaluate feasibility of the intervention and given the nature of the vulnerability of study participants, all participants involved (adults at the end of life, family members and hospice staff) participated in the intervention for an average of 48 days, with no follow-up beyond that. Recruitment was non-coercive and potential participants were not approached by the researcher, instead hospice staff approached and introduced the project to the participants. Participants were asked to complete selected patient reported outcome measures (Integrated Palliative Outcome Scale (IPOS) [52], Hospital Anxiety and Depression Scale (HADS) [53] and Personalised Music Assessment Tool [54]) at three different intervals, once before the intervention as a baseline, one on day 3 and one after the final session on day 7. Participants were observed by the researcher three times during the intervention in addition to a qualitative interview. The purpose of the observational component was to observe fidelity to the intervention and to observe the nature of patient and family member interaction/responsiveness to the setting up of, and listening together to the playlist, and to the nature of response to the personalised music intervention. The nature of observation was discreet but not covert, that is, the researcher adopted the role of observer allowing them to sit within the room.

Any effect of the intervention was assessed by the change in outcome in relation to the baseline data. Therefore, participation time was a total of nine hours plus the qualitative interview over the study period. Involvement in the study was approximately 48 days from consent to final contact with the study team and all study interviews were conducted in a private room. We also recorded any changes in medications being used for pain, anxiety or distress during the intervention period.

A topic guide was developed in conjunction with our Patient and Public Involvement partners during the developmental and modelling stage to provide a level of structure to the qualitative interviews (Appendix 1). The guide was based on the research questions and aimed to ensure consistency between participants.

### Participants and setting

Participants were recruited in one hospice setting from April 2019 to March 2020 by clinicians in the participating hospice who received study information and agreed to participate. Eligible participants were (1) hospice inpatient or day hospice patient with family member/friend interested in participating in Playlist for Life (for the purposes of this study, family is defined as whomever the patient identifies as family, including friends, caregivers or faith or spiritual care leaders); (2) English as a first language; (3) cognitively intact and able to consent to intervention; and (4) prognosis > 7 days. Exclusion criteria were (1) significant confusion/delirium, (2) rapid physical deterioration, (3) expressed no interest in taking part in the Playlist for Life intervention and (4) prognosis ≤ 7 days.

### Intervention

Participants recruited from the hospice were inpatients or day care patients (aged 18 and over with English being their first language), with the aim of recruiting an equal number from both participant groups. A staff member and a family member were present when the patient listened to the Playlist. Together with a family member, the participant chose music tracks that they wished to hear, and a unique personal music playlist was set up on a mp3 player. Music covered many different music genres from easy listening to rock. Most participants chose a combination of genres from different decades of their lives. A staff member from the hospice was trained in Playlist for Life 1 day training package (provided by Playlist for Life charity) with a protocol based on Gerdner [29] and facilitated the setting up of the Playlist on the mp3 player. Hospice staff helped participants to create a unique personal music list by facilitating song choice and documenting them. Hospice staff had a key role in providing person-centered music listening intervention and helping with processing of emotions for each participant. An important part of the training is around recognising ‘Red Flag’ songs, songs which bring a negative emotional response. These are not put on the individuals’ playlist as per the Gerdner protocol [29].

The participant and family member engaged in discussion and exploration to discover the autobiographical memories and emotional connections the participant had for the selected songs or pieces of music. A notebook was provided for the dyad to document this dialogue. Gift boxes were provided by the research team to the hospice to present the music player, playlist and notebook to the participants’ family following the participants’ completion of their involvement in the study.

Compilation of the playlist involved 2 activities: (i) gathering music preferences for the unique personal playlist from each participant and, (ii) exploring and discovering autobiographical memories and emotional connections for the songs or pieces of music.

Each activity comes with an estimated time of 1–2 hours (comprising of several short interactions if required depending on fatigue/other symptoms). Aspects and issues arising from the above around ease of use, potential challenges and barriers to setting up Playlist for Life was assessed as part of the feasibility study.

The average length of time participants were in the study was 48 days (range 28–84 days). On each day of the 7-day intervention period participants and family member/friends listened together to 30 min of their own playlist. The average total listening time of the intervention was 3.5 hours over 1 week. The post-intervention interviews with participants and family members were conducted at a suitable time for the dyad following the 7-day listening session.

The setting up of the Playlist and listening sessions were assessed through participant observation and patient reported outcome measures which were completed throughout the 7 days of the intervention period. There were three observed sessions within the intervention period. The discussion of the setting up of the playlist was observed, as well as two listening sessions. Participants were asked to complete the patient reported outcome measures at three different times, one before the intervention as a baseline, one on day 3 and one after the final session on day 7.

### Feasibility and acceptability of Playlist for Life

To evaluate feasibility and acceptability, the patient reported outcome measures (PROMs) included Integrated Palliative Outcome Scale (IPOS) [52], Hospital Anxiety and Depression (HAD) Scale [53] and Personalised Music Assessment Tool [54].

Integrated Palliative Outcome Scale (IPOS) is a tool which measures the symptoms and concerns of people receiving palliative care. This tool can be used in clinical care, audit, research and training. IPOS has 17 items which measure the following dimensions in advanced illness: physical, psychological, emotional, spiritual and provision of information and support [52].

Hospital Anxiety and Depression Scale (HADS) assesses anxiety and depression in a general medical population of patients. It includes 14 questions to evaluate depression and anxiety, seven of the questions relate to depression and seven relate to anxiety and takes 2–5 min to complete [53].

Personalised Music Assessment Tool (PMAT) includes 5 items which capture the impact of the playlist on pain, personal care, eating and drinking, communication, medication and mood [54].

In view of the potential burden to participants, the PROMS were chosen on the basis of stakeholder assessment and are validated for use with people at the end of life. They were chosen based on ease and speed of administration as well as the suitability of the patient reported outcome measures to answer the research questions.

Qualitative data were collected from participant observation sessions and qualitative semi structured interviews post-intervention. These were audio-recorded, transcribed and analysed thematically. Listening sessions were observed by the same researcher. Questions were developed by the research team with the aim of eliciting an understanding of participants perceptions in participating in the Playlist for Life intervention, experienced barriers related to the intervention, and suggestions for refinement of the intervention processes (Appendix 1).

In addition, the researcher kept a journal throughout data collection documenting each playlist choosing session/listening session 1/listening session 2 and after the qualitative interviews. Process issues related to the qualitative components of the study were documented in the journal reflect on any pertinent issues for future sessions. This included researcher observations and subjective experience relating to the researcher role in the study. Field notes were also kept within the diary, providing additional contextual data and an audit trial of the data collection.

### Recruitment and retention

The recruitment and retention rates were calculated at the hospice based on: the number of participants assessed as clinically eligible to take part; the number of eligible participants who consented to take part; the number of participants who completed the trial; the number of those who declined to take part and reason for doing so; and, the number of people who dropped-out or failed to complete the study along with reasons for leaving [55].

### Sample size

A formal sample size calculation was not performed as the aim of this study was to evaluate the feasibility and acceptability of the Playlist for Life intervention for adults at the end of life, family members and hospice staff [56]. Feasibility studies are not expected to have large sample sizes to power statistical null hypothesis testing [57].

### Data analysis

Descriptive statistics (mean, median, percentages) were calculated for PROM scores and demographic data. The participant observation and interview data were analysed using thematic analysis as outlined by Braun and Clarke [58]. Thematic analysis can be applied within different theoretical frameworks. In this study the method was described as “contextualist”, which covers the ground between essentialism and constructionism [58]. Adopting this approach reports on the experiences, meanings and reality of participants in relation to the study intervention. This approach also enables a critical assessment of how the shared social context of the Playlist for Life intervention exerts influence on meaning. Our aim was to develop a rich thematic description across the data set to identify significant themes. Based on the analysis, decisions to add, change, delete or reword items were conducted by two of the authors. We have adhered to the consolidated criteria for reporting qualitative research (COREQ) using their comprehensive 32-item checklist to support rigour and transparency in the design and conduct of the qualitative research component and the subsequent reporting of the qualitative research findings [59].

### Ethical considerations

NHS ethics (IRAS) ethical approval were sought and granted (Ref:^19^/WS/0077). Clinical governance, research management and access approval were granted from the hospice Clinical Governance group and Chief Executive.

Participation was voluntary and written informed consent was obtained for all participants before the intervention. Participants had a minimum of 24 hours to read the information sheet and decide whether to take part prior to consenting. The preservation of confidentiality and anonymity of participants were ensured. Demographic data, informed consent and data from the documents observation and interviews were transcribed and saved on an encrypted device and stored in a secure drawer in a locked room at the University of Glasgow.

## Results

### Participant characteristics

A total of *n* = 18 participants were enrolled (*n* = 6 patients, *n* = 6 family, *n* = 6 staff), with two participants withdrawing from the study, one patient was withdrawn on medical advice, one patient withdrew themselves due to deteriorating health, one family member participated but was not consented so their data were not included. Therefore, the study started with 18 participants and ended with 15 participants (*n* = 4 patients, *n* = 5 family, *n* = 6 staff). Most participants were female (*n* = 10) with a sample median age of 59 years (range 34–82). All patient participants (100%) had a cancer diagnosis as their primary illness. The average experience in the hospice service for staff was 6 years (ranging from 6 months to 11 years). All family members taking part were identified by participants as the person who mattered most to them. Detailed participant demographic data are shown in Table 1.

**Table 1:** Participant demographics

Patient		Age	Diagnosis		Date of diagnosis		Date of hospice admission/attendance	
1		58	Brain tumour		12/2014		14/4/2019	
2		82	Oesophageal cancer		2/2018		31/5/2018	
3		70	Small cell lung cancer		3/2018		29/5/2018	
4		56	Renal cancer		11/4/2018		24/7/2019	
5		60	Gastric cancer		28/8/2018		24/7/2019	
6		82	Renal cancer		06/2019		12/9/2019	
**Family**		Age	Gender		Relationship to patient		Number of years known to patient	
1		58	Female		Wife		42	
2		51	Female		Daughter		51	
3		–	–		–		–	
4		74	Female		Mother		56	
5		57	Male		Brother		57	
6		84	Male		Husband		71	
**Staff**	Age	Gender	Job role	Hospice service	Years in post	Years qualified	Previous palliative care experience	Previous playlist for life experience
1	38	Female	Registered Nurse	Day	4.5	17	Yes	Yes
2	56	Female	Healthcare assistant	Day	11	n/a	No	No
3	60	Female	Healthcare assistant	Day	4	n/a	Yes	Yes
4	50	Female	Registered Nurse	Inpatient	7	24	Yes	Yes
5	37	Female	Non clinical	Inpatient	9	n/a	No	Yes
6	34	Female	Registered Nurse	Inpatient	6 months	10	Yes	Yes

^*^Participant numbers were randomly assigned

### Participant feedback

Statements from all three participant groups have illustrated the desire for Playlist for Life to continue and be rolled out to more people. All participants were also supportive of the way we delivered Playlist for Life in this trial. Overall, it was thought to be an improvement to the existing delivery of Playlist for Life within the hospice. Prior to the structured Playlist for Life intervention being introduced, the work had been undertaken in a much more informal and ad hoc basis, reliant on day to day availability of appropriate team members and relatives. The use of a more formal template and reflection added a level of robustness and reliability to ensure the process was completed in a more timely and comprehensive manner.

The inclusion of a family member was met positively and has been recognised by staff within the unit as a positive and important aspect of the intervention, both during the song selection process, but also throughout the playback sessions and as patients become frailer towards end of life. This is a clear illustration that our methods work and are acceptable to participants, their families, and staff and can integrate well with inpatient hospice care as it is currently provided.

Many of the family members also reported that taking part in this project has had benefits for them. Taking part in the project enhanced their perceived quality of life and the chosen songs included a shared memory that both the participants and their family member had a connection to. In addition, some participants emphasised that the benefits were simply the enjoyment of listening to music. They also recounted remembering happier times and reminiscing about their past as well as remembering poignant memories and occasions. Music can offer different things to different people, but it is important to remember regardless of what music evokes for that person that they perceived that it was having a positive effect on them and their relationship with their family member.

Through the interviews, it was clear that all participants wanted to see the good and positive in Playlist for Life. They wanted it to succeed, wanted it to work for participants and families. This demonstrates that Playlist for Life is a popular intervention, and one people are willing to invest their time and effort into. Furthermore, according to all participants the methods used in this feasibility study were suitable for implementation in a future randomised controlled trial and developed as a standardised intervention package.

The length of interviews ranged from 5 to 23 min, with an average length of 12 min and listening session lengths ranged from 10 to 75 min. Common themes generated from the interviews were: legacy for future, precious memories and living in the moment. The summarised themes with example quotes are shown in Appendix 2.

### Legacy for future

This theme determined how the participant and family member found the experience of setting up their own playlist and what this means for them in terms of legacy. The staff interviews in particular highlighted and focused on Playlist for Life being something that could have a lasting effect for both the patient while alive and their family when the patient has died. Indeed, particular examples raised from staff include the benefit for the family of having a Playlist for Life in the final hours of their loved ones life. Three sub themes including (i) legacy for people, (ii) legacy for others, and (iii) life after hospice/project were identified under this theme.

### Legacy for people

According to the findings, legacy for people is an essential facilitator to help individuals for making their life enjoyable and dying peacefully. Participants have highlighted how using playlist has made their life more joyful while approaching end of their life: **‘**I feel really chuffed that I’ve managed to pick between eight and ten songs that are all so different, going over a large part of my life. I never thought I’d be able to do that before so it makes me really pleased and I’ll enjoy listening to it for as long as I can.’ (Patient participant 5)

The Playlist for life intervention also allowed participants to share their deepest thoughts and *feelings* while approaching their end of life journey: ‘I think it’s a way of opening up for … especially, for men, because west of Scotland men aren’t the best at opening up with their feelings and to be able to put it across in a way where it’s music that’s bridging that gap … it’s lovely. It’s just lovely, and I think it’s a great legacy.’ (Staff 3). Similarly, another participant said: ‘they might not be that, sort of person, to, you know, pour all their emotions, but one song could tell the family exactly how they're feeling’ (Staff 2).

### Legacy for others

The findings in this study have shown that leaving a legacy for others via the Playlist for Life can be an important tool to help individuals to cope with their bereavement and grief after the death of a loved one. In the words of one participant: ‘But they’ve got even closer through doing this and I think that’s invaluable, that the relationship they’ve now got and then when the daughter does eventually lose her dad, that she will have this and the memories they’ve made, it’s been fantastic.’ (Staff 1)

Another participant said that: ‘I think it’s definitely something that’s beneficial for the families for when the patient dies and it’s something that they have to remind them of that special person’ (Staff 5).

### Life after hospice/project

Comments from participants were favourable regarding use of the intervention in the future. One participant narrated the following: ‘I hope it goes forward and it helps many, many people in the future.’ (Patient participant 2 talking about the intervention). Another participant highlighted: ‘ … it’s not just a wee thing that happens one off, it does continue with them, and they still thank you. They’re still listening to it.’(Staff 3)

The Playlist for Life intervention can also be important to enhance participants psychological well-being and provide a pre- and post-bereavement support for family members. As one participant said: ‘*..well, prior to this I would go home, and I’d be sitting miserable. But I’m going home now, and I would … I’ve had a cheerful afternoon and I even spend some time listening to other tunes, which I think she would enjoy, later on at night. I’m on the computer … what songs she’s picked, what she … you could add to it. So, I believe we’ll be carrying on after this.’ (Family 6)*

### Precious memories

The Playlist for Life intervention helped participants to remember their precious memories in the past. It provided an opportunity to disclose and reflect on positive and negative life experiences, and a history about their whole story from the birth to death.

One participant stated: ‘It left me feeling good from the point of view that … it was a memory of many things that … what it did for me was it began to bring me back to some of the nice times in life and the fun times, but also that was tinged with sadness because I then began to realise that although,` you went through life and you saw life as a path and a path of fun and enjoyment that that doesn't really exist without some sadness being in there also.’ (Patient participant 2)

The Playlist for Life intervention helped to remind the participants of their life in relation to their family. One participant mentioned that the playlist recalled the precious memories related to family members: ‘Each time I listened to it. It just made me think about [brother] more and as what we are, brother and sister, and not just, you know … back then we were just growing up in that house. But we were a family, we were bonded together and that music kind of emphasised that, I think.’ (Patient participant 2)

This intervention can also help individuals to cope with psychological distress at the end of life by realising their positive personal qualities. In the words of another participant:

‘Songs that reminded him of parts of his life when he was strong and you know, that will maybe help him at this stage in his life.’ (Family 2)

### Living in the moment

Another finding of this study was that the Playlist for Life intervention enabled participants to deal with daily problems by helping them to live in the moment. For instance,

‘It makes me feels good, and I enjoy listening to the music that I had forgotten … I've been looking forward to my wee visits and things, because sometimes you feel neglected here when the house is empty, but we've got a project so it keeps your mind going, do you know what I mean sort of thing, but the core things are still there, but music helps. I'm taking more notice of it now.’ (Patient c 1)

It also contributed to the experience of a joyful life and making life ‘worth living’ by helping to live in the present for people approaching the end of life. As one participant said: ‘It just brightens up your life, doesn't it. Songs you listen to it. Makes you happy and want to dance and you can't, but, anyway, I just sit in my wheelchair and dance but, aye, it makes you happy, a happy feeling.’ (Patient participant 6) In the words of another participant: ‘It makes you sit and spend a wee bit of time together and think about different things and enjoy the pleasure of music together.’ (Family 2)

In addition, according to our findings, the Playlist for Life intervention can help individuals to cope with the challenges of being ill by maintaining normality for people at the end of the life. One participant stated: ‘I think, it helps to keep everything as normal as possible.’ (Staff 3)

### Outcome measure scores

With regard to the outcome measures (Integrated Palliative Care Outcome Scale, Hospital Anxiety and Depression Scale, Personalised Music Assessment Tool) most patients (*n* = 4) completed all measures after the intervention. However, two participants (patient participant 3 and 4) did not complete measures day 3 and 7. All baseline questionnaires bar patient participant 4 were filled out with the researcher present. Patient 4 filled out the baseline PROMS themselves. Day 3 questionnaires, bar patient participant 5, were filled out by participants themselves or with help from hospice staff (patient 6). In addition, day 7 questionnaires were filled out by participants themselves or with the researcher present. These were filled in more completely as the researcher conducted the second listening observation session on day 7 and was able to prompt the participant to fully fill in questionnaires. Data from the participants indicated that outcome measures scores did not change to a great extent between the baseline score, day 3 score and day 7 score. Details around percentage changes in scores along the outcome measures is shown in Tables 2, 3, 4, 5, and 6

**Table 2:** Hospital Anxiety and Depression Scale results

Patient	Baseline HADS score		Day 3 HADS score		Days 7 HADS score	
	Anxiety	Depression	Anxiety	Depression	Anxiety	Depression
1	13	8	11	1/1	11	7
2	18	18	18	14	16/18	14
3	11	6	–	–	–	–
4	5	4	–	–	–	–
5	5/9	5/12	10	8	9	12
6	1	0	–	–	0	0

All scores are out of 21 unless indicated, e.g. 16/18 = 16 points out of a possible 18, where patients have not completed a question

**Table 3:** Integrated Palliative care Outcome Scale (IPOS)–baseline results

Patient	Main problems/concerns in the past week	Symptom score (out of 40)	Other symptoms Score (each out of 4)	Q3-Q9 score (out of 28)	How questionnaire was completed
1	None	12	Headaches, 2	9	With help from relative
2	Illness, Family, Panic attacks	22	None	21	On my own
3	Advancement of cancer diagnosis, Side effects of illness.	32	None	11	With help from staff
4	None	17	None	13	With help from relative
5	Illness, symptoms getting worse	13	None	9	With help from staff
6	None	10	None	0	With help from staff

**Table 4:** Integrated Palliative care Outcome Scale (IPOS)–IPOS day 3 results

Patient	Main problems/concerns in the past week	Symptom score (out of 40)	Other symptoms Score (each out of 4)	Q3-Q9 score (out of 28)	How questionnaire was completed
1	Balance, trying to keep active, Bereavement	2	None	10	On my own
2	Family, irrational thinking, fear of the unknown	19	Worry, 4. Family, 4. Fear of unknown, 4.	19	On my own
3	-	–	–	–	–
4*	-	–	–	–	–
5	My condition, family, dealing with the general situation.	14	None	13	With help from staff
6	None	9	None	4	With help from staff

^*^Patient 4 too lethargic to complete PROMS on day 3

^*^Patient 3 was withdrawn on medical advice

**Table 5:** Integrated Palliative care Outcome Scale (IPOS)-IPOS Day 7 Results

Patient	Main problems/concerns in the past week	Symptom score (out of 40)	Other symptoms Score (each out of 4)	Q3-Q9 score (out of 28)	How questionnaire was completed
1	None	4	None	10	On my own
2	Family, fear of the unknown, concern over a purchase	17	Fear of the unknown, 3. My illness, 3.	18	On my own
3	–	–	–	–	–
4*	–	–	–	–	–
5	Worry over symptoms, family, what will happen next.	14	None	12	With help from staff
6	Going home or to a Care home when leaving the hospice	12	None	5	With help from staff

^*^Patient 4 too lethargic to complete PROMS on day 7

^*^Patient 3 was withdrawn on medical advice

**Table 6:** Personalised Music Assessment Tool results

Patient	Baseline	Day 3			Day 7		
		Pre	During	Post	Pre	During	Post
1	17	–	–	–	26	26	26
2	24	18/24	–	–	22	17/24	22
3	20	–	–	–	–	–	–
4*	22	–	–	–	–	–	–
5	24	27	29	28	24	26	26
6	26	–	–	–	25	29	26

All scores are out of 29 unless indicated, e.g. 17/24 = 17 points out of a possible 24 where a patient has not answered every question

^*^Patient 4 too lethargic to complete PROMS on day 3 and day 7

PROMs were straightforward for staff to understand. One highlighted barrier to completing paperwork in the inpatient hospice was time. The PROMs were completed most effectively when researcher was present, sometimes assisting. The Integrated Palliative Care Outcome Scale was completed the most fully. There were 4 out of 14 occasions when the Hospital Anxiety and Depression Scale was not filled out fully. Again, there were four out of 14 occasions when the Personalised Music Assessment tool was not filled out comprehensively, Participants said the paperwork was straightforward, one patient noted it was repetitive. No significant changes were noted in medication use for participants during the playlist intervention period (Table 7).

**Table 7:** Drug changes

	Drug	Route	Day 1 Dose	Day 3 Dose	Day 7 Dose
Patient 1	Epilim	Oral	500 mg	500 mg	500 mg
	Levothyroxine	Oral	150 mcg	150 mcg	150 mcg
	Omeprazole	Oral	20 mg	20 mg	20 mg
Patient 2	Simvastatin	Oral	40 mg	40 mg	40 mg
	Omeprazole	Oral	40 mg	40 mg	40 mg
Patient 3	Alogliptin	Oral	25 mg	25 mg	25 mg
	Amitriptyline	Oral	5 mg	5 mg	5 mg
	Diazepam	Oral	2 mg	2mg	2 mg
	Cetrizine	Oral	10 mg	10 mg	10 mg
Patient 5	morphine	SC	10 mg	10mg	10 mg
	Midazolam	SC	2 mg	2 mg	2 mg
	Omeprazole	Oral	40 mg	40 mg	40 mg
	levothyroxine	Oral	100 mcg	100 mcg	100 mcg
Patient 6	Enoxaparin	SC	40 mg	40 mg	40 mg
	Clopidogrel	Oral	75 mg	75 mg	75 mg
	Lansoprazole	Oral	30 mg	30 mg	30 mg
	Prednisolone	Oral	2 mg	2 mg	2 mg

## Discussion

The aim of this study was to evaluate the feasibility, acceptability and practicality of using Playlist for Life for adults at the end of life, family members and hospice staff in a hospice inpatient unit setting in Scotland. Given that many people with life-threatening illnesses will continue to have increased needs that could be met through the provision of palliative care as their condition progresses, it is important that interventions that may facilitate and shape future care are used during a time when input from individuals approaching the end of life is possible [1].

The Playlist for Life intervention enabled participants to leave their loved ones a legacy which could help bereaved individuals to cope with their grief after their loved ones death. It also enabled people who are nearing end of life to feel more generative (the strive to create or nurture things that will outlast individuals). According to Erikson’s (1963) theory of psychosocial development, leaving a positive legacy before death is an important part of the generativity for individuals [60].

Participants indicated that the intervention enabled their legacy through a personalised playlist that they can share with their loved ones, now and in the future after they die. In addition, the Playlist for Life intervention was found to be a useful tool to help participants relate their lives in relation to their personalities and families, and to help them recognise their positive personal qualities. In this way, participants were able to look back at their life with a sense of satisfaction. In Erikson’s theory it has been highlighted that individuals who have ego integrity are able to face the end of their life with no regrets and can accept the death as an integral and natural part of life [60]. This intervention can also facilitate individuals’ search for meaning, especially while facing a life-threatening disease. Facilitating and supporting patients in pursuit of meaning at the end of life is also important to improve psychological well-being in people with life-threatening disease [61].

The findings of our feasibility study also demonstrated that the Playlist for life intervention could be likened to creating a person-centred ‘time capsule’ for participants—a journey to their past and present and even looking into the future after they have died. This concurs with previous evidence that highlighted that music worked like “acoustic reminders of childhood memories” and encouraged individuals to open up, to travel back and forth between the past, the present and the future [62]. This travel also enabled them to share their deepest thoughts and *feelings* and complete unfinished business, thus providing critical information about their plans for the future, while approaching their end of life journey [63]. Staff found that participants really welcomed the opportunity to ‘speak to their family’ in terms of the playlist. Also, in the study families were ‘gifted’ a booklet with the songs chosen and why these were chosen. This allowed people to tell their families some things, including loving and personal memories, they were not able to verbalise. In addition, previous evidence from a randomised, controlled trial demonstrated that music intervention fostered feelings of resilience and increased expression of emotions [64].

Our study results demonstrated that the intervention was enjoyable, brought mixed emotions both happy and sad, and that it enabled participants to be closer to their family members by sharing old and creating new memories. Participants indicated that the intervention helped them to live in the moment and they felt relaxed while enjoying the pleasure of music, reducing symptoms like anxiety and distress. Music affects the release of neurotransmitters which are responsible for the induction of feelings of happiness and for the reward system in the brain [65]. Hence, all of these feelings of participants may be explained by the release of the neurotransmitters such as dopamine and serotonin, which play an important role in reducing stress [40]. In addition, previous clinical studies have demonstrated the psychological effects of music on people who have a life-threatening illness, with significant results in reducing anxiety, stress, depression, thus improving quality of life, mood and psychological well-being [32–39]. A study by Garabedian and Kelly (2020) reported that music intervention was successful at transporting listeners away from their present reality into an absorbing, fulfilling, and enjoyable ‘haven’ [34]. This evidence demonstrates that music has an important role in creating a joyful environment in individuals lives. According to the literature creating a joyful environment can facilitate living in the moment (i.e. listening to music, gardening, building a shed, reading a newspaper). Living in the moment for people nearing end of life is important to help them cope with the uncertainty of life by providing dignified person-centred care [66]. Focusing on simple pleasure and not worrying about the future enables people to carry out their daily routines, and helps them set realistic goals [66–68]. Even though most participants experienced greater well-being while listening to music, given their emotional vulnerability, the surfacing of sad memories can still occur and may be distressing. Therefore, it is important to not leave a person to listen to their music alone and to provide a safe environment [18].

This study sought to assess the feasibility of an intervention to improve future care for people at the end of life, as well as their willingness to engage in such a study. The study has yielded good indicators for this group of participants as well as a desire to take part. It showed that the Playlist for Life intervention was well received by people with life-threatening illness, their family members and hospice staff. All participants felt that Playlist for Life could help people at the end of life and their family. In addition, feedback was favourable regarding use of the intervention in the future. The method and outcome measures proposed were appropriate and acceptable for participants, did not pose any significant difficulties and also provided results that could provide useful information if used in a future larger-scale randomised controlled trial.

In this feasibility study, the intervention approach was based on the selection of a playlist by people at the end of life and their family member together. This approach provided closer relationships among participants and their families, while engaging in discussion and exploration to discover the autobiographical memories and emotional connections the patient had for the selected songs or pieces of music. This process also helped them to create their life history through a unique personal music playlist as a special legacy. According to literature, the act itself of choosing music together allows a deep connection to be quickly established [69]. The act of the patient and family member choosing music makes use of the potential of the individual, focusing on ‘noticing, acknowledging and making use of the individual’s resources via the fostering of a collaborative relationship’ Indeed, this approach including the collaborative therapeutic relationship is a core component to delivering person-centred care for people at the end of life [69].

Feasibility evaluation is an essential part of developing complex interventions, preventing problems that could undermine the conduct or acceptability of future evaluation and informing the parameters of the future evaluation design [70]. The findings of this feasibility study highlight the possibility of implementing the Playlist for Life intervention for adults at the end of life, and their family members and prove high compliance with the intervention protocol and evaluation.

## Limitations and strengths

This study has several strengths: first, the mixed-method design using a multiple baseline design and qualitative study. Second, the protocol was refined through stakeholder consultation. Third, discussions with academics and clinical experts helped to prevent the systematic bias from a single researcher. In addition, the qualitative interviews provided additional insights into other aspects not easily assessed by quantitative outcome measures.

We acknowledge the limitations of this study. The limitations of our feasibility study are that it is limited by the small sample from one hospice setting and only included participants who have cancer. Participants might have wanted to see the good and positive features of the intervention as they were volunteers and were recruited by hospice staff initially. The study did not have the aim of generating an assessment of effectiveness, although useful indicators have been yielded about the suitability of outcome measures for a future study. Beyond a pilot study, a large-scale randomised controlled trial would seek to confirm generalisability of results.

## Conclusion

This study has shown that Playlist for Life is feasible and acceptable as a person-centred intervention for adults at the end of life, family members and hospice staff in a hospice inpatient setting in Scotland. The feasibility findings show that it is worth proceeding to a larger scale study that will provide more detailed analysis of findings and widen the use of the outcome measures to provide greater pre- and post-intervention evaluation. The study has produced some evidence that may inform and enhance the care of people who have a life threating disease to address the needs of patients and their families and deliver a quality person-centred palliative care in hospice settings. Furthermore, Playlist for Life is currently being used in the intervention study site with staff members working with the intervention reporting positive experiences about its utility and benefits for people and their families.

## Data Availability

The datasets during and/or analysed during the current study are available from the corresponding author on reasonable request.

## Appendix

Appendix 1 Sample interview questions

1. What is the most acceptable and appropriate format for patient/carer delivery of the Playlist for Life intervention within the context of palliative and end of life hospice-based care?

2. What are the most relevant patient reported outcome measures for assessing participant experience of Playlist for Life?

3. How will we conduct the participant observation? Do our research questions for the qualitative component provide rich, descriptive data on participant experience?

4. Based on the above, can we develop a standardised intervention package that is suitable for implementation in a future randomised controlled trial?

5. What are the recruitment and retention rates for patents and family members based on one hospice inpatient unit?

6. Does participant observation data point to the benefits of Playlist for Life and is there sufficient fidelity to the manual for Playlist for Life implementation in the participating hospice unit?

7. Do staff and family views of the intervention indicate acceptability to proceed with Playlist for Life?

8. Does Playlist for Life integrate well within the current service model and care pathways in the participating hospice?

9. Is there sufficient evidence to support the design of a full cluster trial?

Appendix 2 Overarching themes for 'Playlist for Life'

**Table:**

Overarching themes	Details	Sample quotes
**Legacy for Future**	This theme determined how patients and family members found the experience of setting up their own playlist and what this means for them in terms of legacy.	*‘so he’s now died peacefully knowing that in a way that he’s left an apology for her and she’s got that. So she’s got peace and comfort afterwards.’ (Staff 1)* *‘I think it’s definitely something that’s beneficial for the families for when the patient passes away and it’s something that they have to remind them of that special person’ (Staff 5)*
**Precious memories**	The Playlist for Life intervention helped participants to remind their precious memories in the past. It provided an opportunity to disclose and reflect on positive and negative life experiences, and a history about their whole story from the birth to death. This intervention also facilitated help individuals to cope with psychological distress at the end of life by realising their positive personal qualities.	‘It left me feeling good from the point of view that there were many … it was a memory of many things that … what it did for me was it began to bring me back to some of the ice times in life and the fun times, but also that was tinged with sadness because I then began to realise that although you went through life and you saw life as a path and a path of fun and enjoyment that that doesn't really exist without some sadness being in there also.’ (Patient 2) ‘Songs that reminded him of parts of his life when he was strong and you know, that will maybe help him at this stage in his life.’ (Family 2)
**Living in the moment**	The Playlist for Life intervention contributed to the experience of a joyful life and making life 'worth living' by helping to live in the present for people approaching the end of life. It also provided to deal with daily problems by helping them to live in the moment and maintain normality.	*‘It just brightens up your life, doesn't it. Songs you listen to it. Makes you happy and want to dance and you can't, but, anyway , I just sit in my wheelchair and dance but , aye, it makes you happy, a happy feeling.’ (Patient 4)* *‘I think, it help to keep everything as normal as possible.’ (Staff 3)*
